# Gut microbiota-generated metabolite, trimethylamine-*N*-oxide, and subclinical myocardial damage: a multicenter study from Thailand

**DOI:** 10.1038/s41598-021-93803-7

**Published:** 2021-07-22

**Authors:** Vichai Senthong, Songsak Kiatchoosakun, Chaiyasith Wongvipaporn, Jutarop Phetcharaburanin, Pyatat Tatsanavivat, Piyamitr Sritara, Arintaya Phrommintikul

**Affiliations:** 1 Cardiovascular Unit, Department of Medicine, Faculty of Medicine, Khon Kaen University, Khon Kaen, Thailand; 2 Department of Biochemistry, Faculty of Medicine, Khon Kaen University, Khon Kaen, Thailand; 3 Khon Kaen University International Phenome Laboratory, Northeastern Science Park, Khon Kaen University, Khon Kaen, Thailand; 4 Cardiovascular Unit, Department of Medicine, Faculty of Medicine, Ramathibodi Hospital, Mahidol University, Bangkok, Thailand; 5 Cardiovascular Unit, Department of Medicine, Faculty of Medicine, Chiang Mai University, Chiang Mai, 50200 Thailand

**Keywords:** Evolution, Biomarkers, Cardiology

## Abstract

Plasma Trimethylamine-*N*-oxide (TMAO), a gut microbiota metabolite from dietary phosphatidylcholine, is mechanistically linked to cardiovascular disease (CVD) and adverse cardiovascular events. We aimed to examine the relationship between plasma TMAO levels and subclinical myocardial damage using high-sensitivity cardiac troponin-T (hs-cTnT). We studied 134 patients for whom TMAO data were available from the Cohort Of patients at a high Risk of Cardiovascular Events—Thailand (CORE-Thailand) registry, including 123 (92%) patients with established atherosclerotic disease and 11 (8%) with multiple risk factors. Plasma TMAO was measured by NMR spectroscopy. In our study cohort (mean age 64 ± 8.9 years; 61% men), median TMAO was 3.81 μM (interquartile range [IQR] 2.89–5.50 μM), and median hs-cTnT was 15.65 ng/L (IQR 10.17–26.67). Older patients and those with diabetic or hypertension were more likely to have higher TMAO levels. Plasma TMAO levels correlated with those of hs-cTnT (r = 0.54; p < 0.0001) and were significantly higher in patients with subclinical myocardial damage (hs-cTnT ≥ 14 ng/L; 4.48 μM vs 2.98 μM p < 0.0001). After adjusting for traditional risk factors, elevated TMAO levels remained independently associated with subclinical myocardial damage (adjusted odds ratio [OR]: 1.58; 95% CI 1.24–2.08; p = 0.0007). This study demonstrated that plasma TMAO was an independent predictor for subclinical myocardial damage in this study population.

## Introduction

Trimethylamine-*N*-oxide (TMAO) is a gut microbiota metabolite from dietary phosphatidylcholine (PC) which present in red meat, egg yolks and liver. Increasing data support the role TMAO in the pathogenesis of cardiovascular disease (CVD)^1–5^. Importantly, elevated plasma TMAO levels are correlated with future risk of adverse cardiovascular events, increased CVD prevalence, number of diseased coronary vessels and atherosclerotic burden of coronary artery disease (CAD)^2,6,7^.

High sensitivity cardiac troponin is a sensitive and specific biomarker of myocardial damage and has been used in the clinical diagnosis of myocardial infarction in acute coronary syndrome (ACS)^8^. Assays to detect circulating high-sensitivity cardiac troponin (either T or I) allow for the robust detection of very low troponin concentrations, an indicator of subclinical myocardial damage (SMD) in asymptomatic patients^9,10^. Several studies have shown a link between SMD and future risk of adverse cardiovascular events in stable patients with and without CVD, particularly in the general population^9–14^.

Several studies have demonstrated the mechanisms underlying the link between elevated plasma TMAO and CVD pathogenesis/risk of cardiovascular events. We thus sought to examine the relationship between plasma TMAO levels and high-sensitivity cardiac troponin T (hs-cTnT) in stable patients at high risk of cardiovascular events.

## Results

### Patient characteristics

Overall baseline characteristics of the 134 patients in the study population are shown in Table 1. The mean age was 64 years, 61% were men, 40% had diabetes, 123 (92%) had established atherosclerotic disease (EAD), and 11 (8%) had multiple risk factors (MRFs). Median TMAO was 3.81 μM (Interquartile range [IQR] 2.89–5.50 μM), and hs-cTnT was 15.65 ng/L (IQR 10.17–26.67).

**Table 1:** Baseline characteristics of study participants.

Variables	Overall (N = 134)	Subclinical myocardial necrosis (SMN)		P value
		Yes N = 77	No N = 57	
Age, (year)	64.05 (8.9)	63.6 (8.4)	64.6 (9.5)	0.53
Male sex, (%)	82 (61.2)	56 (72.7)	26 (45.6)	0.001
BMI, (kg/m^2^)	24.62 (4.19)	24.85 (4.02)	24.31 (4.43)	0.47
Systolic BP, (mm Hg)	131.49 (19.45)	132.74 (18.89)	129.79 (20.22)	0.39
Diastolic BP, (mm Hg)	73.1 (9.52)	73.91 (9.61)	72.0 (9.36)	0.25
Heart rate (beat per min)	73.6 (12.97)	74.16 (13.8)	72.82 (11.82)	0.56
Coronary Artery Disease (%)	123 (91.8)	71 (92.2)	52 (91.2)	0.99
History of myocardial infarction (%)	99 (73.9)	60 (77.9)	39 (68.4)	0.22
Percutaneous coronary intervention (%)	100 (74.6)	61 (79.2)	39 (68.4)	0.16
Coronary artery bypass graft surgery (%)	4 (3.0)	2 (2.6)	2 (3.5)	0.99
Diabetes mellitus (%)	53 (39.6)	33 (42.9)	20 (35.1)	0.36
Hypertension (%)	67 (50.0)	38 (49.4)	29 (50.9)	0.86
Current smoker status	8 (6.0)	5 (6.5)	3 (5.3)	0.99
hs-cTnT (ng/L)	15.65 (10.17–26.67)	24.4 (17.9–37.2)	9.05 (6.3–11.5)	< 0.001
Dyslipidemia (%)	53 (39.6)	75 (97.4)	56 (98.2)	0.99
eGFR (ml/min/1.73 m^2^)	72.4 (51.7–88.55)	73.6 (49.8–88.1)	69.8 (54.4–88.7)	0.67
**Medication**				
Aspirin or Clopidogrel (%)	126 (94)	74 (96.1)	52 (91.2)	0.28
ACEI or ARB (%)	85 (63.4)	54 (70.1)	31 (54.4)	0.06
Statin (%)	131 (97.8)	75 (97.4)	56 (98.2)	0.99
Beta-blockers (%)	109 (81.3)	62 (8.5)	47 (82.5)	0.78
TMAO (μM)	3.81 (2.89–5.50)	4.48 (3.45–6.14)	2.98 (2.31–4.23)	< 0.0001
Established CVD	123 (91.8)	71 (92.2)	52 (91.2)	0.99

Values are mean ± SD, %, or median (inter-quartile range).

*ACEI* angiotensin converting enzyme inhibitors, *ARB* angiotensin-receptor blocker, *BMI* body mass index, *BP* blood pressure, *eGFR* estimated glomerular filtration rate, *hs-cTnT* high-sensitivity cardiac troponin T, *TMAO* trimethylamine *N*-oxide, *CVD* cardiovascular disease.

Baseline characteristics were stratified according to presence of SMD. Overall, there were 77 (57%) patients with SMD (hs-cTnT ≥ 14 ng/L). Those with evidence of SMD were more likely to be male, and a slightly higher percentage used angiotensin converting enzyme inhibitor/angiotensin receptor blocker (ACEI/ARB). By contrast, history of hypertension, diabetes, EAD status, and renal function did not differ by SMD status. Interestingly, SMD was significantly associated with higher TMAO levels (4.48 μM, [IQR: 3.45–6.14] vs 2.98 μM [IQR: 2.31–4.23]; p < 0.0001).

### Plasma TMAO levels and subclinical myocardial damage

**Figure 1:**
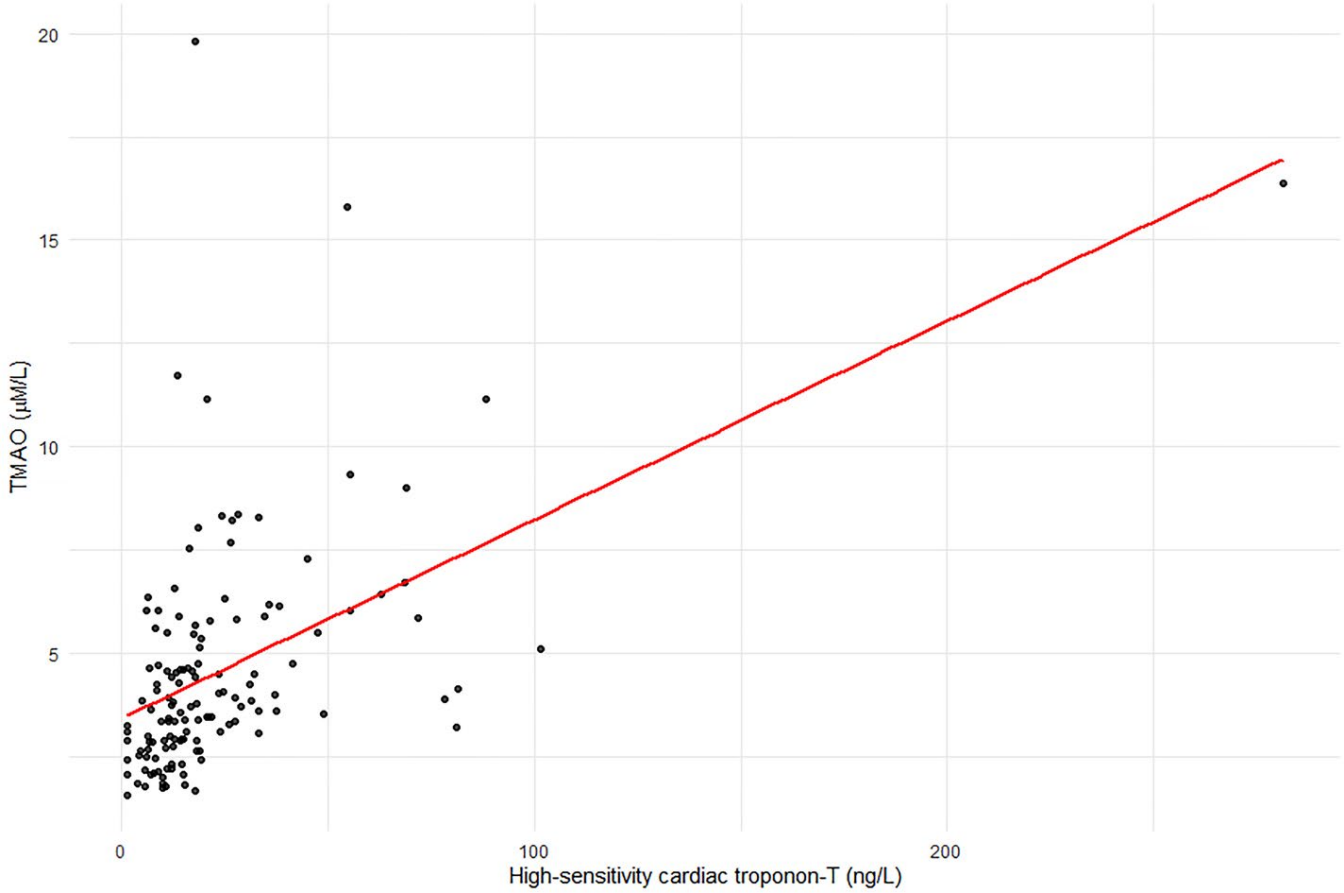
Correlation between plasma TMAO levels and high-sensitivity cardiac troponin-T.

**Figure 2:**
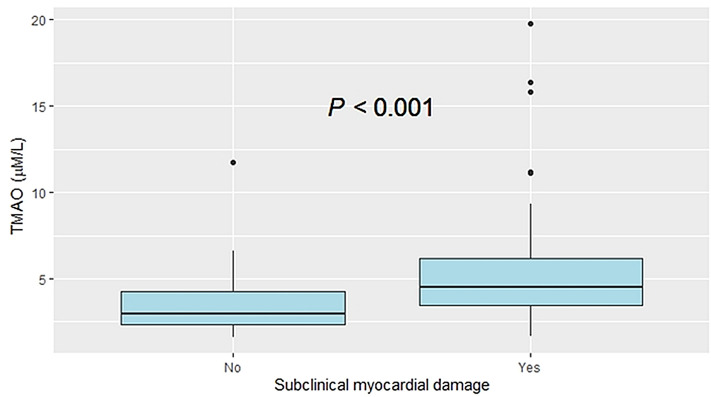
Relationship between plasma TMAO levels and subclinical myocardial damage.

Plasma TMAO was strongly correlated with hs-cTnT (Pearson correlation; r = 0.54, p < 0.0001) (Fig. 1), and TMAO levels were significantly higher in patients with evidence of SMD (Fig. 2). In univariate logistic regression analysis, plasma TMAO levels, male, EAD, estimated glomerular filtration rate (eGFR), and ACEI/ARB use were significantly associated with SMD (Table 2).

**Table 2:** Univariate and Multivariate Logistic Regression for Prediction of Subclinical Myocardial Damage (hs-cTnT ≥ 14 ng/L).

	Univariate Analysis		Multivariate Analysis		
	OR (95% CI)	P value	Adjusted OR* (95% CI)	P value	VIF
TMAO	1.84 (1.22–1.92)	< 0.0001	1.58 (1.24–2.08)	0.007	1.05
Age	0.99 (0.94–1.04)	0.67	0.99 (0.94–1.04)	0.67	1.48
Male	3.18 (1.54–6.56)	0.02	3.68 (1.55–7.20)	0.004	1.22
BMI	1.03 (0.95–1.12)	0.47	0.69 (0.02–10.5)	0.74	1.39
Dyslipidemia	1.12 (0.47–2.76)	0.78	0.89 (0.36–2.16)	0.79	1.06
Diabetes mellitus	0.72 (0.36–1.46)	0.36	0.96 (0.38–2.40)	0.78	1.22
Hypertension	1.01 (0.99–1.03)	0.39	1.27 (0.44–3.71)	0.66	1.09
Smoking	0.8 (0.83–3.49)	0.78	1.14 (0.19–7.13)	0.88	1.10
Established CVD	0.54 (0.21–1.36)	0.19	1.45 (0.31–6.74)	0.63	1.08
eGFR	0.99 (0.98–1.01)	0.18	0.98 (0.96–1.0)	0.89	1.45
Statin	0.75 (1.32–16.9)	0.75	–	–	–
ACEI/ARB	0.4 (0.19–0.83)	0.014	2.03 (0.87–4.87)	0.10	1.01
Antiplatelet	0.42 (0.09–1.84)	0.25	–	–	–

*Adjusted for age, male, BMI, dyslipidemia, diabetes mellitus, hypertension, smoking, established.

CVD, eGFR and ACEI/ARB use.

*TMAO* trimethylamine *N*-oxide, *ACEI* angiotensin converting enzyme inhibitors, *ARB* angiotensin-receptor blocker, *BMI* body mass index, *eGFR* estimated glomerular filtration rate, *hs-cTnT* high-sensitivity cardiac troponin T, *CVD* cardiovascular disease.

Elevated TMAO remained a significant predictor of SMD after multivariate logistic regression analysis, which adjusted for traditional risk factors (age, male, smoking, dyslipidemia, diabetes, hypertension, body mass index and eGFR), EAD status, and ACEI/ARB use (adjusted odds ratio [OR] 1.58; 95% CI 1.24–2.08, p = 0.0007; Table 2). By contrast, EAD, eGFR, and ACEI/ARB use were no longer significant predictors of SMD.

We further examined the association of TMAO and SMD using multiple logistic regression analysis, adjusting for any variable with a p-value < 0.3 according to univariate analysis including age, male, eGFR, EAD status, and ACEI/ARB use. Interestingly, elevated TMAO remained a significant predictor of SMD (adjusted OR 1.55; 95% CI 1.23–2.04, p = 0.0007; Table 3).

**Table 3:** Adjusted odds ratios (OR) using variables with P-value < 0.3 based on univariate analysis.

	Subclinical myocardial damage (hs-cTnT ≥ 14 ng/L)		
	OR (95% CI)	P value	VIF
**Adjusted**			
TMAO	1.55 (1.23–2.04)	0.0007	1.01
Age	0.97 (0.84–1.12)	0.61	1.38
Male	3.24 (1.40–4.36)	0.001	1.15
eGFR	0.98 (0.97–1.00)	0.11	1.15
ACEI/ARB	2.08 (0.93–4.76)	0.08	1.00
Established CVD	1.40 (0.72–1.48)	0.74	1.06

Abbreviations as in Table 2.

To our knowledge, this is the first report of elevated plasma TMAO as an independent predictor of SMD (quantified as hs-cTnT ≥ 14 ng/L) in stable patients at high risk of cardiovascular events (123 [92%] with EAD and 11 [8%] with MRFs). The prevalence of SMD in our study cohort was 57%, despite our including stable patients without evidence of ACS at presentation. The key finding of our study is the strong significant association between fasting plasma TMAO levels and SMD. Furthermore, elevated TMAO was an independent predictor of SMD, even following adjustment for traditional risk factors, EAD status, and ACEI/ARB use.

TMAO, a gut microbiota-generated metabolite, plays an important role in global metabolism. Recent reports have shown that increased levels of plasma TMAO are associated with adverse cardiovascular outcomes and have a direct mechanistic link to the development of CVD^1–5^. This is consistent with several other recent studies in subsets of stable patients with elective coronary angiography, peripheral artery disease, diabetes mellitus, chronic kidney disease, CAD, history of heart failure (HF), and even those without CVD^1–3,5,7,15^.

Interestingly, another previous study found elevated TMAO to be associated with risk of major adverse cardiac events among patients with suspected ACS who were initially negative for cardiac troponin T (< 0.1 ng/mL)^16^. Moreover, elevated TMAO can predict the presence of coronary atherosclerotic burden and complexity as defined by SYNTAX score and diffuse lesion characteristics^6^. These findings were independent of traditional risk factors and were validated by independent cohorts, which were reviewed in a recent meta-analyses^17^. However, the association between TMAO and evidence of SMD as indicated by higher hs-cTnT, has not been well explored. This was the first study to show elevated plasma TMAO to be an independent predictor of SMD. This is consistent with the gut microbiota TMAO pathway being mechanistically linked with the development of atherosclerosis and adverse prognosis.

Detection of circulating hs-cTnT is associated with myocardial damage and is a recommended diagnostic criterion for myocardial infarction in ACS^18^. There is evidence that low hs-cTnT concentration in subjects without clinical setting of ACS indicates an increased risk of future cardiovascular events and may predict poor prognosis^10,19^. Low hs-cTnT concentration is a useful biomarker to stratify patients by risk level (both in stable CVD patients and in the general population)^9,11,13^. In stable patients with chronic HF or stable CAD, higher hs-cTnT is associated with increased risk of cardiovascular events^10,12^. Previous studies have shown that low hs-cTnT is associated with higher incidence of cardiovascular events, total mortality, and cardiac abnormalities (e.g., left ventricular hypertrophy, left atrial enlargement, and silent ischemia) in the general population^9,12^.

In our study cohort, we defined SMD as hs-cTnT ≥ 14 ng/L, which is the 99^th^ percentile cut-point for diagnosis of myocardial infarction. Importantly, although we included stable patients without evidence of ACS at presentation, elevated plasma TMAO was associated with SMD. Moreover, while hs-cTnT is often higher in patients with declining of renal function^20^, elevated plasma TMAO remained an independent predictor of SMD even after adjusting for eGFR and other potentially confounding factors. Therefore, it is conceivable that SMD may occur in with elevated plasma TMAO. This may be a clue to understanding the underlying mechanism through which elevated plasma TMAO is linked to enhanced risk of CVD and poor prognosis.

Previous studies have consistently demonstrated that the TMAO pathway is linked mechanistically to multiple cardiovascular and metabolic processes with CVD pathogenesis including the development of atherosclerotic plaque, promotion of adipose tissue inflammation, alteration in macrophage and endothelial cells, plasma lipid abnormalities, insulin resistance, obesity, and enhanced platelet hyperactivity and thrombosis risk, all of which contribute to SMD due to troponin leak^1–6,17,21–23^.

TMAO may exhibit direct biological activity in modulating platelet hyperactivity, which increases the risk of thrombus formation^16,23^. Several recent studies have demonstrated that flavin-containing monooxygenase 3 (FMO3), the major host enzyme responsible for converting gut microbiota-generated trimethylamine (TMA) into TMAO, is an important regulator of sterol metabolism, prevents reverse cholesterol transport, and is linked to the development of atherosclerosis^21,24^. By contrast, FMO3 knockdown mice had decreased circulating TMAO levels and attenuated atherosclerosis plaque formation^25^. Moreover, recent studies have found that a high fat diet plus 0.2% TMAO promotes adipose tissue inflammation in mice, which be related to SMD^26^.

Our findings shed light on a potential pathophysiological contribution of gut microbiota in the development of atherosclerosis and to adverse prognosis in patients with high atherosclerotic risk. Further study is needed to determine whether therapeutic strategies that reduce plasma TMAO levels can also improve prognosis in these patients.

### Study limitations

This study had the following limitations: first, only patients with available TMAO data were included, which may have led to selection bias. Second, despite all subjects being recruited from the outpatient clinic after fasting for at least 8 h, we could not exclude the potential for dietary intake of PC or TMAO within 24 h before blood sampling. Third, because plasma TMAO and hs-cTnT were measured at only one point in time, we were unable to evaluate the prognostic value of changes in these indicators. Finally, a relatively low number of patients with MRFs were included in this study.

## Conclusions

Fasting plasma TMAO is an independent predictor of SMD in patients with high atherosclerotic risk. These findings are consistent with numerous previous studies that have demonstrated a link between elevated plasma TMAO and atherosclerosis pathogenesis and cardiovascular risk. Further study into the mechanism by which elevated TMAO leads to SMD is warranted.

## Subjects and methods

The Cohort Of patients at a high Risk of Cardiovascular Events—Thailand (CORE-Thailand) registry is a prospective, multicenter, longitudinal cohort study of Thai patients with high atherosclerotic risk. This includes patients aged 45 years or older with EAD and those with MRFs, as detailed in previous publications^27^.

We studied 134 patients for whom TMAO data were available. This study was approved by the Khon Kaen University Ethics Committee for Human Research (HE611011) and was conducted in accordance with the Declaration of Helsinki.

### Laboratory testing

Informed consent was obtained from all patients and fasting blood samples were collected using EDTA tubes at the time of hospital visit. These were then immediately processed and frozen at − 80 °C until analysis. Plasma TMAO levels were determined using an NMR spectrometer at 400 MHz (Bruker, USA). The Carr-Purcell-Meiboom-Gill (CPMG) pulse sequence was employed to obtain spectra (recycle delay-90°-t1-90°-tm-90°-acquisition) over 64 scans with four dummy scans. Quantification was achieved using a concentration of a known reference signal (in this case TSP) to determine the TMAO concentration. hs-cTnT was measured using a high-sensitivity (5^th^ generation) assay on a Roche Cobas e411 platform (Roche Diagnostics, Basel, Switzerland). We defined SMD as hs-cTnT ≥ 14 ng/L, as this is considered the upper limit (the 99^th^ percentile) of the normal range in the healthy population^19^. eGFR was calculated using the modification of diet in renal disease (MDRD) equation.

### Statistical analysis

Continuous data are presented as means (standard deviation) or medians (interquartile range) and compared with a student’s t-test or non-parametric test (Mann–Whitney U Test) as appropriate. Categorical variables are presented as numbers (%) and compared between groups using a chi-square test. The correlations between hs-cTnT and plasma TMAO-associated factors were analyzed using the multicollinearity index. Comparisons among three or more groups were evaluated using one-way analysis of variance (ANOVA) or the Kruskal–Wallis test depending on whether or not the distribution was normal. Univariate and multivariate logistic regression analysis was used to determine independent predictors of SMD. Variables entered into the multivariate model included traditional risk factors (age, male, smoking, dyslipidemia, diabetes, hypertension, body mass index, and eGFR), ACEI/ARB use, and EAD status. All analyses were performed using R version 4.0.3. A p value < 0.05 was considered statistically significant.

## Abbreviations

CAD: Coronary artery disease
CVD: Cardiovascular disease
TMAO: Trimethylamine-*N*-oxide
hs-cTnT: High-sensitivity cardiac troponin T
SMD: Subclinical myocardial damage
